# Effect of Shear Strain Rate on Microstructure and Properties of Austenitic Steel Processed by Cyclic Forward/Reverse Torsion

**DOI:** 10.3390/ma12030506

**Published:** 2019-02-07

**Authors:** Zhimin Zhang, Qingshan Dong, Bo Song, Hong He, Linjiang Chai, Ning Guo, Bingshu Wang, Zhongwen Yao

**Affiliations:** 1 School of Materials and Energy, Southwest University, Chongqing 400715, China; zzm816816@email.swu.edu.cn (Z.Z.); hong1979@swu.edu.cn (H.H.); 2 Department of Mechanical and Materials Engineering, Queen’s University, Kingston ON K7L3N6, Canada; qingshan.dong@queensu.ca; 3 College of Materials Science and Engineering, Chongqing University of Technology, Chongqing 400054, China; yaoz@queensu.ca; 4 Department of Materials Science and Engineering, Fuzhou University, Fuzhou 350108, China; bswang@fzu.edu.cn

**Keywords:** gradient structure, shear strain, strain rate, deformation-induced martensite transformation, torsion

## Abstract

In this work, commercial AISI 304 stainless steel rods were subjected to cyclic forward/reverse torsion (CFRT) treatments at low-speed and high-speed torsion at room temperature. Microstructures in the core and surface layers of the CFRT-treated samples were systematically characterized. Results show that the CFRT treatment can introduce martensite phase on the surface of the rods via strain-induced martensitic transformation. High-speed twisting is more effective in inducing martensite in the surface layer compared to low-speed twisting. During the stretching process, the overall strain-hardening behavior of the gradient material is related to the content of its gradient defects. Higher gradient martensite content results in a higher surface hardness of the material, but less overall tensile properties. The effect of twisting speed on torsion behavior and the strain-hardening mechanisms in tensile of the gradient structured steels was also addressed.

## 1. Introduction

A common goal of structural materials research is to prepare materials with high strength and high toughness. However, most strategies that can effectively increase strength may sacrifice ductility, which is the so-called “strength–ductility trade-off” phenomenon [1,2]. Controlling the distribution and organization of defects is considered to be one of the effective ways to solve this problem [3]. Increasing the defect content can increase the strength of the material, and controlling the distribution of defects is expected to improve the plasticity of the material. 

Nano-grained (NG) metals are usually brittle. However, the NG metal film which is produced by the coarse-grained (CG) metal substrate to produce a gradient structure shows high strength and high toughness—a 10 times higher yield strength and a tensile plasticity comparable to that of the CG substrate [4]. Metal surface nanocrystallization via surface mechanical treatment is considered to be a very effective method for preparing high-performance gradient structural materials [5,6]. In recent years, more and more studies have demonstrated that introducing gradient defects, such as gradient dislocations [7], gradient lamellar dislocation substructures [8], gradient twins [9,10], gradient grains [6,11,12], gradient textures [1,13], and even gradient second phase particles [14,15] into materials, can effectively overcome the strength–ductility trade-off. In recent years, Guo et al. [8,16] have pointed out that torsion processing is not only a common material fatigue test method, but an efficient strategy to prepare a quantitative controlled gradient structure for rod-shaped parts. High-performance gradient structural materials can be prepared by controlling torsion path [14,17], torsion speed [18], and the combined annealing process [19,20]. Recently, we have developed a new method to introduce a gradient of martensite phase in austenitic steels, with a volume fraction increasing from core to surface, via free-end torsion (FET). The treated samples simultaneously showed high strength and high toughness. Moreover, it was also found that cyclic forward/reverse torsion (CFRT) is more effective than unidirectional-torsion (UT) in inducing martensitic transformation, and can enhance the gradient distribution of the martensite phase [14]. The CFRT treatment provides an approach to develop high-strength good-ductility steel and other alloyed metals. However, it is considered that CFRT is affected by many factors, such as twisting speed, twisting angles, and number of cycles. Among them, for the CFRT treatment, the torsion speed is considered to be the main factor controlling the gradient martensite content and distribution. However, there have not been relevant research reports yet. In this paper, AISI304 stainless steel rods were processed by CFRT technology at different torsion speeds, and the microstructure was systematically characterized. The effect of shear strain rate on torsion behavior, microstructure, tensile properties, and fracture mechanisms were investigated. The strain-hardening mechanisms in the tensile of gradient structured steels were also addressed.

## 2. Experimental Studies

**Figure 1:**
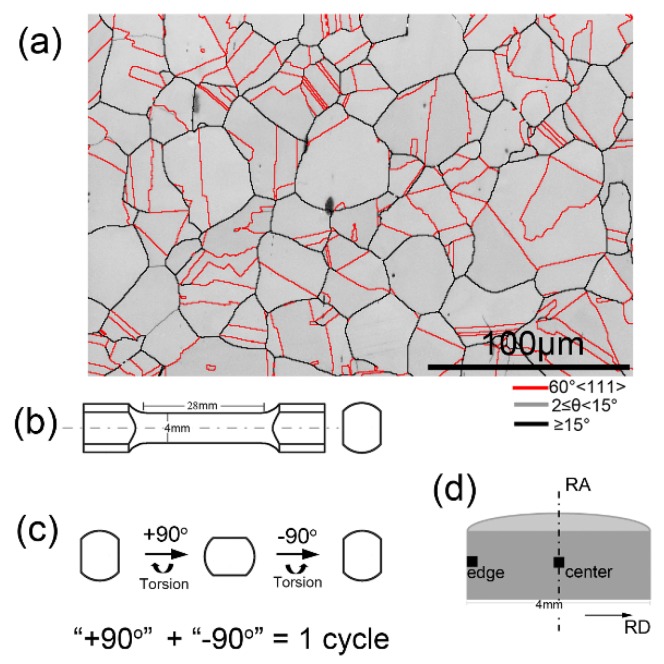
Microstructure before torsion (**a**), sample size of torsion processing (**b**), processing schematic (**c**), and locations of microstructure characterization (**d**).

Commercial AISI 304 stainless steel (304 SS) rods with a diameter of 10 mm were selected as the starting materials. The chemical composition is Fe–18.56Cr–8.25Ni–1.17Mn–0.68Si–0.30Cu–0.23Mo–0.08C (wt %). The starting rods were treated at 1050 °C for 0.5 h, and then water-quenched to obtain a fully austenitic structure with an average grain size of about 36.0 μm in diameter (Figure 1a). Dog bone-shaped samples with gauge dimensions of 4 mm in diameter and 28 mm in length were cut from the as-quenched rods (Figure 1b). Cyclic forward/reverse torsion (CFRT) was performed on a free-end torsion machine at room temperature. As shown in Figure 1c, rotating forward by 90° plus reverse by 90° is defined as one cycle. Two twisting speeds, including low-speed (at 10°/min) and high-speed (at 1800°/min) rotating were employed, respectively. After torsion, the uniaxial-tensile test was carried out at room temperature and a strain rate of 1.0 × 10^−3^ s^−1^. Hardness along the radius direction of the sample was measured using a Vickers indentation tester (HVS-1000) at a load of 200 g and a loading time of 10 s.

Scanning electron microscope (SEM, Zeiss Sigma HD, Zeiss, Dresden, Germany) equipped with electron backscattered diffraction (EBSD, AZtech Max2, Oxford Instruments, London, UK) and scanning/transmission electron microscope (STEM/TEM, Tecnai Osiris, FEI Company, Hillsboro, OR, USA) were employed to characterize the microstructure of the twisted samples. Microstructural characterization was carried out on the longitudinal sections of the samples and the characterized locations were near the core and surface layer (see Figure 1d). Step sizes of 30–200 nm for EBSD mapping were used. Prior to EBSD, the specimens were ground and electropolished with an electrolyte of 10% perchloric acid and 90% methanol at −20 °C. The TEM specimens were ground to about 60 μm thick first, and then thinned by twin-jet electropolishing (TenuPol-5, Struers, Ballerup, Denmark) using the same electrolyte at −40 °C. 

## 3. Results and Discussion

### 3.1. Microstructure Evolution

**Figure 2:**
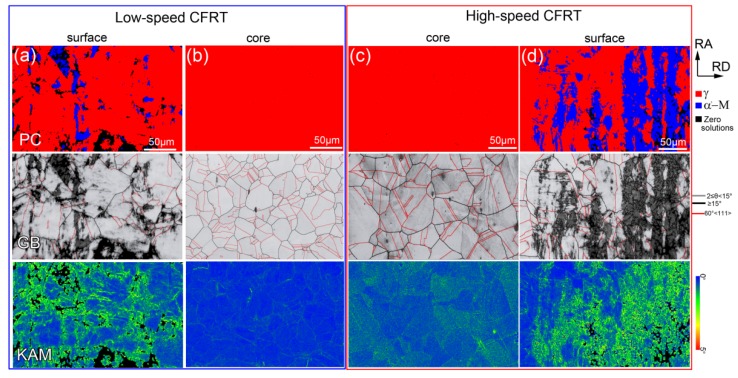
Electron backscattered diffraction (EBSD) results showing phase constitution (PC) map, grain boundary (GB) map, and kernel average misorientation (KAM) map and of the twisted samples at different strained layers. (**a**) and (**b**) are surface and core layers of the sample twisted for 100 cycles at 10°/min, respectively. (**c**) and (**d**) are core and surface layers of the sample twisted for 100 cycles at 1800°/min, respectively.

Figure 2 presents the EBSD results of the samples twisted for 100 cycles with different torsion speeds. Figure 2a and b show phase constitution (PC), grain boundary (GB), and kernel average misorientation (KAM) maps obtained in the surface and core layers of the sample twisted at low speed, while Figure 2c and d display the microstructure in the core and surface layers of the sample twisted at high speed. It is well known that the KAM value calculated by the Hough-based analysis of EBSD data is a reflection of geometrically necessary dislocation (GND) density, and a high KAM value indicates a high dislocation density [21,22]. It can be seen that the microstructure in the core layer of the two samples is similar, showing similar grain sizes and twin densities (see GB map), low dislocation density (see KAM map), and no martensite (see PC map), which is almost identical with the microstructure before torsional deformation (see Figure 1a). That is, regardless of the torsion speed, the microstructure and defect content of the core layer after torsion does not change much compared with the untwisted state (see Table 1). Similar phenomena have been found in magnesium alloys and copper during unidirectional torsion deformation [8,16]. It has been reported that because of the nature of the torsion process, the torsion strain is linearly proportional to the distance from the core [23]. The main reason is that the strain is in a gradient distribution during torsion deformation, the surface is subjected to a high amount of plastic deformation, while small, or even no, plastic deformation occurs in the core, resulting in the microstructure in the core region maintaining the pre-torsion state [19,20,24]. After torsion, martensite (α′-M) with a body-centered tetragonal (BCT) structure is observed on the surface. Moreover, compared to the low-speed torsion, more α′-M is observed on the surface layer of the high-speed torsion, as shown in Table 1. Strain-induced martensite transformation, generally from face-centered cubic (FCC) structure γ → hexagonal close-packed (HCP) structure (ε-M) → α′-M, occurs in austenitic steels during cold deformation [25,26,27]. It has been reported that the formation of gradient α′-M is due to the gradient shear strain and strain rate of torsion [14].

**Figure 3:**
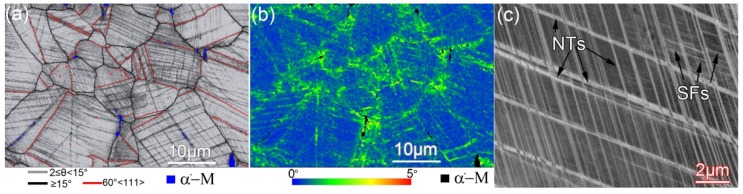
EBSD results showing deformation twins and stacking faults (SFs) at the surface layer of the sample twisted for 100 cycles at 10°/min. (**a**) GB map, (**b**) KAM map, and (**c**) ECCI image.

**Figure 4:**
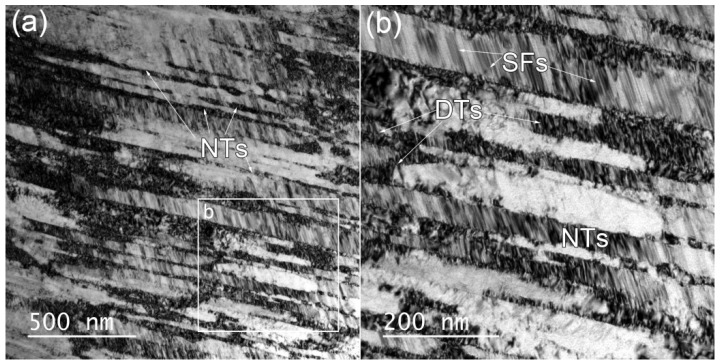
Transmission electron microscope (TEM) bright field (BF) images showing nanotwins (NTs): (**a**) dislocation tangles (DTs) and (**b**) stacking faults (SFs) in the surface of the sample twisted for 100 cycles at 10°/min.

During cold deformation, metastable austenite is prone to strain-induced martensitic transformation, and due to its low stacking fault energy, it is prone to twinning during deformation [28]. However, the EBSD results show that the number of twins on the surface layer of the sample does not increase or even decrease after torsion (see GB maps in Figure 2a,d. Figure 3 shows high magnification EBSD and ECCI (electron channeling contrast imaging) images observed on the surface layers of the sample twisted at the low speed. As can be seen, in fact, after the low-speed torsion, a large number of nanotwins (NTs) are generated inside the grains on the sample surface. The thickness of the NTs is extremely thin (less than 80 nm, see Figure 3c, so that most NTs are not recognized by the EBSD mapping. In addition, a large number of stacking faults (SFs) are distributed between the NTs. The TEM observations also confirmed this, as shown in Figure 4. In addition to SFs, a large number of dislocation tangles (DTs) are observed at the twin junctions (see Figure 4b). The dislocations can tangle with thin twin lamellae and SFs during cold deformation, and have been observed in 304L stainless steel during tensile deformation [28]. It is considered that due to the presence of these DTs, there is a higher KAM value near the twin boundaries in the KAM map (see Figure 3b). It can be concluded that for high-speed torsion, the surface layer contains a higher martensite structure, while for low-speed torsion, the surface layer contains more NTs.

### 3.2. Effect of Twisting Speed on Torsion Behavior

**Figure 5:**
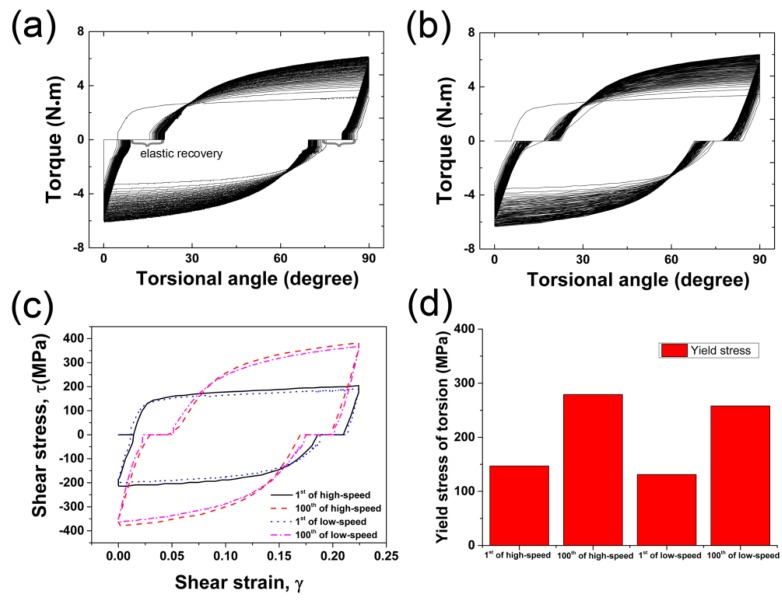
Microstructure before torsion (**a**), sample size of torsion processing (**b**), processing schematic (**c**), and locations of microstructure characterization (**d**).

The measured torque, plotted as a function of torsion angle after CFRT treatment at different torsion speeds, is shown in Figure 5. Clearly, whether it is a fast twist or a slow twist, as the number of reciprocating cycles increases, the area of the closed loop composed of the torque and the twist angle gradually increases, indicating an increase in mechanical work after each cycle of the CFRT. Additionally, it should be noted that although the torsion path is designed to be “forward twisting 90° + reverse twisting 90°”, the experimental results show that only in the first cycle is the actual twist angle of the sample close to 90°, and from the second cycle to the 100th cycle, the actual twist angle is only about 75°. It has been reported that for torsion deformation, elastic recovery occurs during the unloading process of torsion [29,30]. On the other hand, there is a gap between the sample end and the torsion chuck mold of the free-end torsion in this study. The larger the gap, the greater the elastic recovery during torsion deformation. It is considered that due to the elastic recovery during the reverse loading, the actual torsion angles are less than the designed angles. 

Figure 5c shows the shear stress–strain curves of the CFRT-processed sample. The shear stress–strain curves of the outer part are calculated based on the torque-twist angle data using Equation (1) [31]:

(1) $$\tau =\frac{3T}{2\pi R_{0}^{3}},\;\gamma =\frac{R_{0}}{l_{0}}\varphi$$

where *τ* and *γ* are shear stress and shear strain of torsion, *T* and *φ* are torque and twist angle of the outer part of the rod sample, *R*_0_ and *l*_0_ are the initial radius and gauge length of the rod sample, respectively. As can be seen, the torsional yield strength of the high-speed twisted sample is higher than that of the low-speed twisted sample. That is to say, the deformation resistance increases with the increase of torsion speed. Steels are known as being very sensitive to strain rate, and there is a rise of the yield stress when the strain rate increases [32,33]. As the strain rate (tensile) increases, the ultimate stress, fracture stress, and strain-hardening exponent increase, while the ultimate strain and fracture strain decrease noticeably [34]. For diffusion-controlled dislocation slip during plastic deformation, the strain rate dependence of the steady-state flow stress is generated by dislocation recovery controlled by spontaneous annihilation of dislocation and thermal activation process [35]. Faster torsion results in lower dislocation recovery rates and higher flow stresses. 

### 3.3. Strain-Hardening Behavior during Tension

**Figure 6:**
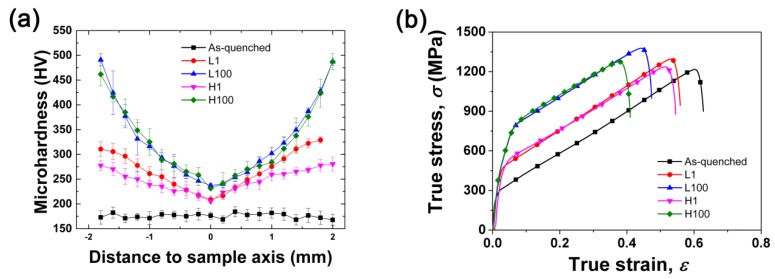
Mechanical properties of various samples: (**a**) Vickers hardness plotted as a function of the distance from the sample axis; (**b**) tensile stress–strain curves.

Figure 6a displays the microhardness distribution from core to surface in various samples before and after torsion. Whether it is slow torsion or rapid torsion, after CFRT, the microhardness distribution is transformed, from a uniform distribution before torsion to a gradient distribution, from the core to the surface layer. Clearly, the hardness value is dependent on the torsional strain. The core has a low hardness, while the surface has a high hardness. The gradient distribution of hardness is the result of the gradient distribution of microstructure. The generation of NTs, DTs, or α′-M particles can hinder dislocation slip and enhance the materials by boundary strengthening or dispersion strengthening [14]. The gradient distribution of these defects creates a gradient of hardness. Moreover, for the microhardness distribution, the gradient characteristics of the high-speed torsion are more remarkable than the low-speed torsion. This is because at the surface of the sample, the high-speed torsion sample contains more strain-induced α′-M than the slow-speed torsion sample. For austenitic stainless steels, the hardening effect of the strain-induced martensite is higher than that of dislocations or twins.

Figure 6b exhibits the tensile curves of the samples along the torsion axis after various CFRT treatments. The detailed mechanical properties are measured and listed in Table 2. It can be seen that the tensile yield strength is very sensitive to torsional deformation and the material strength increases significantly after one cycle of twisting. Moreover, the yield strength of the sample subjected to high-speed torsion processing is always slightly higher than that of the low-speed processed sample, whether it is one cycle of twisting or 100 cycles. After the torsion processing, the uniform elongation (UE) decreases significantly. In the case where the number of twisting cycles is the same, the UE of the high-speed processed sample is slightly lower than that of the low-speed processed sample.

**Figure 7:**
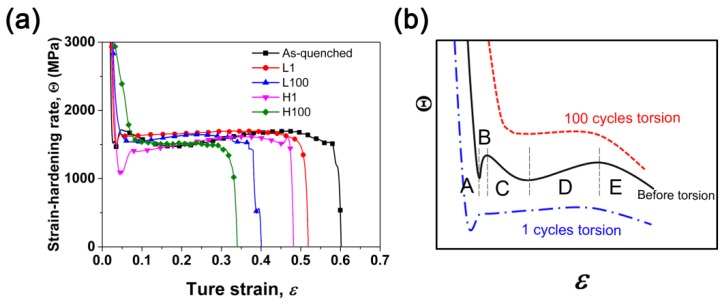
Strain-hardening response of various samples: (**a**) experimental data; (**b**) schematic diagram showing typical strain-hardening curves of FCC materials with low stacking fault energy. The strain-hardening rate *Θ* is calculated as *Θ* = *dσ*/*dε*.

The strength, plasticity, and ductility of materials are closely related to their work-hardening behavior. Figure 7a shows the strain-hardening characteristics of various samples during stretching. For the as-quenched sample with homogenous microstructure, five stages of strain-hardening can be recognized (see Figure 7b). In stage A (*ε* < 0.02), the deformation has just started, and the strain-hardening rate drops sharply. For conventional coarse-grained materials, the deformation in this stage is mainly dominated by dislocation slip with the generation of some SFs [36], as shown in Figure 3 and Figure 4. In stage B (0.02 < *ε* < 0.05), deformation twin nucleates due to multiple twin systems activated during tensile, a large amount of NTs appear in the sample and these twins are harder than the matrix due to the Basinski-hardening mechanism [37], resulting in a significant increase in the strain-hardening rate (stage B). With increasing tensile strain, the strain-hardening rate decreases in stage C, in which dislocation slipping combined with twinning plays an important role. As the tensile strain continues to increase, twinning no longer plays a key role in the deformation process, which could reply more on slip instead. Deformation-induced martensite is formed, and martensite is harder than the austenite matrix, resulting in a slight increase in the work-hardening rate (stage D). At larger strain of stage E, the strain-hardening rate decreases dramatically, resulting in premature plastic instability (necking) [38].

For the samples with only one cycle of CFRT treatment, the gradient defects introduced by pre-twisting are mainly dislocations, SFs, and twins, and the defect content is relatively low. Due to the existence of the pre-induced defects, stage C is not obvious. However, the overall strain-hardening behavior is similar to that of the as-quenched sample, as shown in Figure 7b. For the samples with 100 cycles of CFRT treatment, the content of the pre-induced gradient defects increases remarkably, and a large amount of martensite appears on the surface layer (see Figure 2a,d). The strain-hardening rate transitions directly from stage A to stages D and E, without stages B and C. Therefore, it can be concluded that the overall strain-hardening behavior of the gradient material is related to the content of its gradient defects. When the gradient distribution is significant, the pre-induced gradient defects change the overall strain-hardening behavior.

### 3.4. Fracture Behavior

**Figure 8:**
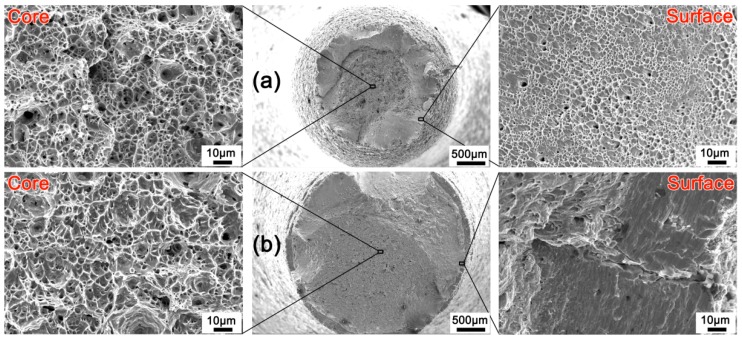
Fracture topography of different samples after tensile test: (**a**) L100; (**b**) H100.

Figure 8 shows the fracture morphology of the L100 and H100 samples. Macroscopically, the cross-sectional shrinkage of the L100 sample is significantly higher than that of the H100 sample, indicating that the L100 sample has better ductility. Generally, on the micro level, the fracture surfaces of ductile materials exhibit dimples generated by dislocation activities at the final fracture stage [39,40], while the brittle fracture surface often has cleavage planes [41], quasi-cleavage planes, or even rock-candy patterns [42]. Both the low-speed and high-speed twisted samples show dimples in the core. This is because the pre-twist introduces fewer defects in the core, whether it is fast twisting or slow twisting. The difference is that the surface layer of the sample twisted at a low-speed has a macro-shear lip that is microscopically composed of slanted dimples (see Figure 8a).

By contrast, the surface of the sample twisted at a high speed has no shear lip, and the outermost layer has a ring-shaped structure with many cleavage planes at the microscopic level (see Figure 3a). This is due to the fact that the H100 sample contains more martensite on the surface layer, and the brittleness of martensite is much greater than that of austenite (see Figure 2a,d). In addition, a microcrack distributed along the diameter direction is observed in the ring-shaped structure, indicating that the crack originates in the surface layer and then spreads to the core during stretching, because more deformation-induced martensite is induced into the surface layer due to the larger strains and higher strain rate at the surface layer [14]. The fracture topography confirms that it is easier to introduce a strain-induced martensite phase on the surface layer by high-speed cyclic forward/reverse torsion.

## 4. Conclusions

In this study, commercial AISI 304 stainless steel rods were CFRT-treated at different speeds. The microstructure and properties of the CFRT-treated samples were characterized and investigated. The main conclusions are as follows:

Compared to low-speed torsion, high-speed torsion sample surfaces have higher martensite content, and lower stacking faults (SFs), nanotwins (NTs), and dislocation entanglements (DTs) in the sample surface. That is, high-speed torsion is more effective in inducing martensite in the surface layer during CFRT treatment.

(1) The torsional yield strength of the high-speed twisted sample is higher than that of the low-speed twisted sample. Faster torsion results in lower dislocation recovery rates and higher flow stresses.
(2) A higher gradient martensite content results in a higher surface hardness of the material, but less overall tensile properties. The tensile strain-hardening behavior of the gradient structured material is related to the content of its gradient defects. With increasing of gradient martensite phase, the strain-hardening rate transits directly from stage A (dislocation slipping-dominated) to stages D (martensite-dominated) and E (necking), without stages B (twinning-dominated) and C (dominated by dislocation slipping combined with twinning).
(3) Both the low-speed and high-speed twisted samples show dimples in the core after the tensile fracture. In the surface layer, the low-speed twisted sample has a macro-shear lip which is microscopically composed of slanted dimples, while the high-speed twisted sample has many cleavage planes instead of macro-shear lips with slanted dimples.

## Figures and Tables

**Table 1:** The length of boundaries per area (μm^−1^) and area percentage of α′-M at core and surface layers of low-speed and high-speed twisted samples calculated from electron backscattered diffraction (EBSD) data.

Defects	Low-Speed		High-Speed	
	Core	Surface	Core	Surface
2° ≤ θ <15°	0.001	0.121	0.002	0.143
θ ≥ 15°	0.020	0.052	0.033	0.069
60° <111>	0.024	-	0.023	-
α′-M	0	5.4%	0	25.3%

**Table 2:** Yield strength (YS), peak strength (PS), and uniform elongation (UE) of various samples.

Samples	YS (MPa)	PS (MPa)	UE (%)
As-quenched	310	1220	60
L1	486	1297	53
L100	781	1381	44
H1	560	1236	51
H100	790	1276	37
